# LncRNA ACTA2-AS1 suppress colon adenocarcinoma progression by sponging miR-4428 upregulation *BCL2L11*

**DOI:** 10.1186/s12935-021-01769-3

**Published:** 2021-04-12

**Authors:** Qingyun Pan, Ying Huang, Yirui Wang, Deke Li, Changjiang Lei

**Affiliations:** 1 Department of Blood Endocrinology, The Fifth Hospital of Wuhan, Wuhan, 430000 Hubei People’s Republic of China; 2 Department of Oncology, The Fifth Hospital of Wuhan, Wuhan, 430000 Hubei People’s Republic of China; 3 Department of Pharmacy, The Fifth Hospital of Wuhan, Wuhan, 430000 Hubei People’s Republic of China; 4 Department of Anesthesiology, The Fifth Hospital of Wuhan, Wuhan, 430000 Hubei People’s Republic of China; 5 Department of General Surgery, The Fifth Hospital of Wuhan, Wuhan, 430000 Hubei People’s Republic of China

**Keywords:** Colon adenocarcinoma, ACTA2-AS1, miR-4428, *BCL2L11*

## Abstract

**Background:**

Long non-coding RNA is considered to be essential to modulate the development and progression of human malignant cancers. And long non-coding RNA can act as crucial modulators by sponging the corresponding microRNA in tumorigenesis. We aimed to elucidate the function of ACTA2-AS1 and its molecular mechanism in colon adenocarcinoma.

**Materials and methods:**

The expression of ACTA2-AS1, miR-4428 and *BCL2L11* in colon adenocarcinoma tissues were detected via qRT-PCR. SW480 and HT29 cells were transfected with shRNA ACTA2-AS1, OE ACTA2-AS1, miRNA mimics of miR-4428, miR-4428 inhibitor, si-*BCL2L11* and over-expression of si-*BCL2L11*. Cell proliferation, colony formation and apoptosis were respectively assessed using CCK-8 assay, colony assay and flow cytometry. Luciferase reporter assay was performed to verify the targets of ACTA2-AS1 and miR-4428. Tumor subcutaneous xenograft mode was constructed to explore tumor growth in vivo.

**Results:**

ACTA2-AS1 was obviously downregulated in human colon adenocarcinoma tissues and colon adenocarcinoma cell lines. Silence or over-expression of ACTA2-AS1 promoted or inhibited cell proliferation and colony formation abilities, and regulated apoptosis. The silence of ACTA2-AS1 resulted in the decrease of Bax and increase of Bal2, while restored in OE ACTA2-AS1 group when compared with the control transfected cells. In addition, luciferase reporter assay revealed that ACTA2-AS1 interacted with miR-4428 and suppressed its expression. miR-4428 could bind to 3ʹ untranslated region of *BCL2L11* and modulated the expression of *BCL2L11* negatively. Knockdown of ACTA2-AS1 and over-expression of *BCL2L11* reversed the biological function that ACTA2-AS1 mediated by knockdown ACTA2-AS1 alone.

**Conclusion:**

Our data demonstrated that ACTA2-AS1 could suppress colon adenocarcinoma progression via sponging miR-4428 to regulate *BCL2L11* expression.

## Introduction

Colorectal cancer (CRC) is one of the most frequently diagnosed tumors with poor prognosis and the most common CRC is colon adenocarcinoma (COAD) [1]. The development and progress of COAD is a multistep process in which accumulating genetic changes can play an important role. Although great progression has been made in surgery, chemotherapy, radiotherapy, and targeted drugs, there is no actual achievements in the overall survival rate of COAD patients [2]. Hence, the investigation of promising therapeutic targets and molecular mechanism involved in the carcinogenesis of COAD remains especially crucial for the early diagnosis, timely treatment, and prognosis.

It is known that long non-coding RNAs (lncRNAs) are RNA molecules with more than 200 nucleotides [3, 4]. Generally, lncRNAs modulate the expression level of targeted genes at the post-transcriptional period [5]. More and more lncRNAs have been found to involve in many aspects of cellular homeostasis, such as angiogenesis, metastasis, cell proliferation, immunity adjustment, genomic stability, and so on [6, 7]. Previous studies also suggest that lncRNAs are involved in the tumorigenesis through multiple mechanisms, such as transcriptional regulation, protein post-translational regulation miRNA regulation and so on [8]. Previous studies also proved that a growing number of lncRNAs play crucial roles in COAD tumorigenesis and development, such as ZDHHC8P1, FOXD3-AS1, and ZEB1-AS [9–11]. Therefore, lncRNAs could be regarded as potential diagnostic and prognostic biomarkers for human cancers.

LncRNA ACTA2-AS1 (ACTA2 Antisense RNA 1) is located at 10q23.31 with five exons [12]. Recent studies revealed that dysregulation of ACTA2-AS1 has been found to be closely related to poor prognosis of several cancers, such as cervical cancer, hepatocellular carcinoma, liver cancer, breast cancer and lung adenocarcinoma [13–15]. According to previous studies, ACTA2-AS1 may play an important role involved in the development of human cancers. However, the role of ACTA2-AS1 in COAD and its underlying molecular mechanisms remains unclear. Our previous studies found an obvious decrease of ACTA2-AS1 expression in both COAD cell lines and COAD tissues, and we presumed that ACTA2-AS1 may act as a crucial regulator in COAD. Therefore, this study is aimed to explore the specific function of ACTA2-AS1 in COAD and the molecular mechanisms involved.

## Materials and methods

### Cell lines and COAD tissues

Normal human colon mucosal epithelial cell line (CCD-18Co) and six COAD cell lines (SW480, HT29, LS174T, HCT116 and DLD-1) were procured from ATCC. The above cells were cultured in RPMI 1640 medium or DMEM (Invitrogen, USA) with 10% fetal bovine serum (Invitrogen, USA) and 1% Penicillin/Streptomycin (Sigma-Aldrich, USA) at 37 °C incubator containing 5% CO_2_. 82 newly diagnosed patients with COAD in The Fifth Hospital of Wuhan were included in the present study. All the experiments were carried out according to the principles of The Fifth Hospital of Wuhan.

### Cell transfection

The design and construction of shRNAs and si-RNA for ACTA2-AS1 and *BCL2L11*, the synthesis of pcDNA 3.1, miR-4428 mimics vector, and the construction of a lentiviral vector overexpressing ACTA2-AS1 and *BCL2L11* were separately conducted by Genechem (Shanghai, China). The transfection was carried out using Lipofectamine 2000 reagent (Invitrogen, USA) according to the guideline of manufacturer. The sequences of genes were as followed:

si-ACTA2-AS1#1: 5′-UAGAUUAUUAUGUCUUCCCAG-3′

si-ACTA2-AS1#2: 5′-UAGUAAAGCAACAUUCUUGGA-3′

si-*BCL2L11*: 5′-UUAAAUAACGUGAACAUGCUG-3′

miR-4428 mimics: 5′-GUUCCUCUGCCCUUGUACCUCG-3′

### RNA extract and quantitative real-time PCR (qRT-PCR) assay

Trizol reagent (TaKaRa, China) was used to extract total RNA according to the manufacturer’s instructions. The PrimeScript RT Master Mix (TaKaRa, China) was used to reverse-transcribe lncRNA and mRNA. The SYBR Premix Ex Taq II Kit (TaKaRa, China) was employed to carry out Real-time PCR. The relative RNA expression was normalized to the expression levels of U6 and GAPDH. The primers used for quantitative PCR were as follows:

ACTA2-AS1:

5′-GTGGTTCTGGTTTGCCTGAT-3′ (forward),

5′-CTGGCCCTGTAACACCAGAT-3′ (reverse);

miR-4428:

5′-GTTCCTCTGCCCTTGTACCTCG-3′ (forward),

5′-GCGCGCGTA ACAGTCTACAGC-3′ (reverse);

*BCL2L11*:

5′-TAAGTTCTGAGTGTGACCGAGA-3′ (forward),

5′-GCTCTGTCTGTAGGGAGGTAGG-3′ (reverse);

U6:

5′-CTCGCTTCGGCAGCACA-3′ (forward),

5′-AACGCTTCACGAATTTGCGT-3′ (reverse);

GAPDH:

5′-TGCACCACCAACTGCTTAGC-3′ (forward),

5′-GGCATGCACTGTGGTCATGAG-3′ (reverse).

### Cytoplasm and nuclear localization

The NE-PER™ Cytoplasmic and Nuclear Extraction Reagents Kit (Thermo Fisher Scientific) was done to confirm the cytoplasmic localization of ACTA2-AS1 in COAD cells. Following the manufacturer’s instructions, the COAD cells nuclear and cytoplasmic constituents were sorted and collected. Afterward, qRT-PCR was used to evaluate the ACTA2-AS1 expression in the nucleus and cell cytoplasm, respectively. GAPDH was used as the cytoplasm localization control while U6 was for the nucleus localization control.

### Cell viability and colony assay

The Cell Counting Kit-8 (CCK-8) (Dojindo, Japan) experiment was used to measure cell viability. COAD cells were seeded into 96-well plates at 37 °C incubator with 5% CO_2_. After transfection, the OD 450 values were measured at 0, 24, 48, and 72 h (h) via CCK-8 assay after incubation for 2 h at 37 °C. Then, the absorbance values were measured on a microplate reader at 450 nm. For colony formation assay, 2000 SW480 and HT29 cells were seeded in 6-well plates and cultured for seven to ten days at 37 °C incubator with 5% CO_2_. Then, the cells were washed twice with PBS (phosphate-buffered saline), fixed with 4% paraformaldehyde (Sinopharm Chemical, China) and stained with crystal violet (Sigma, USA) for 15 min respectively. The clone spots were counted observed under a microscope (Olympus, Japan) with 5 random view fields.

### Apoptotic assay

Apoptotic assay was performed using V-FITC Annexin and PI Apoptosis Detection Kit (Beyotime, China). SW480 and HT29 were fixed in 70% cooled ethanol and stained with Annexin V-FITC and PI for 20 min at room temperature according to the protocol, and then cell apoptosis was detected by flow cytometer.

### Dual luciferase reporter assay

Through starbase 2.0, we found the miR-4428 binding site in ACTA2-AS1 and the downstream target gene of miR-4428 is *BCL2L11*. The 3ʹ-UTR of *BCL2L11* and ACTA2-AS1 containing wild type (wt) and mutant type (mut) reporter vectors were purchased from Beijing TransGen Biotech Co., (Beijing, China). miR-4428 mimics binding sequence was inserted downstream of the firefly luciferase gene in psi-CHECK2 vector to synthesis the *BCL2L11*-wt or ACTA2-AS1-wt and psi-CHECK2-*BCL2L11*-mut or ACTA2-AS1-mut plasmids, respectively. The wt and mut plasmids subsequently were co-transfected into in SW480 and HT29 cells cells with negative control and miR-4428 mimics. After transfection for 48 h, the cells were lysed and the relative luciferase activity was measured via the Dual-luciferase reporter Assay System (Promega, Madison, WI, USA).

### RNA pull-down assay

For RNA pull-down assay, the streptavidin-coated magnetic beads (Life Technologies, CA, USA) were covered by biotinylated ACTA2-AS1 (Bio-ACTA2-AS1) and Bio-Oligo according to its instruction and transfected into l × 10^6^ SW480 and HT29 cells at 50 nM as a final concentration for 48 h. Subsequently, 0.7 mL lysis buffer (5 mM MgClz, 100 mM KCl, 20 mM Tris (pH 7.5), 0.3% NP-40) and complete protease inhibitor cocktail (Roche Applied Science, IN) were added into the cell pellets, then the cell lysates were incubated together with the RNA-tagged beads for the co-immunoprecipitation (Invitrogen, Carlsbad, CA, USA). The RNA–RNA complexes were subsequently collected by centrifugation at 10,000 r for 10 min and then the miR-4428 enrichment level was detected with qRT-PCR analysis.

### Western blotting

Protein was extracted using Radioimmunoprecipitation assay (RIPA, Beyotime, China). Protein samples were separated through 10% sodium dodecyl sulfate-polyacrylamide gel electrophoresis (SDS-PAGE) and then transferred onto PVDF (polyvinylidene fluoride) membranes (Thermo Fisher Scientific, USA). Membranes were blocked in 5% bovine serum albumin (BSA) for 2 h and then incubated with primary antibodies, including anti-Bcl-2, anti- Bax and GAPDH (Cell Signaling Technology, USA) overnight at 4 °C. Next day, after washed with PBST for three times, the membranes were incubated with the HRP-conjugated secondary antibodies (Cell Signaling Technology, USA) at 37 °C for 1 h. Finally, the intensity of the bands was visualized by using enhanced chemiluminescence (ECL).

### Tumor xenografts in nude mice

BALB/c nude mice (16–20 g, 5 weeks of age) were purchased from Shanghai Animal Laboratory Center (Shanghai, China). Cells transfected with sh-NC or sh-ACTA2-AS1 were digested with 0.25% trypsin, diluted in PBS, counted by trypan blue staining, adjusted to a concentration of 1.0 × 10^7 cells/mL, and 0.1 mL (1.0 × 10^6 cells) of this solution was injected hypodermically into the back flank of each mice. Tumor size was calculated every 7 days. Additionally, the present investigation was approved by the ethics committee of The Fifth Hospital of Wuhan.

### Bioinformatics analysis and binding sites prediction

The Cancer Genome Atlas (TCGA, https://www.cureline.com/the-cancer-genome-atlas.html) database from Gene Expression Profiling Interactive Analysis (GEPIA, http://gepia.cancer-pku.cn/) was used to explore the expression of ACTA2-AS1 in COAD patients. The potential miR-4428 binding sites to 3ʹUTR of *BCL2L11* was predicted by Starbase (http://starbase.sysu.edu.cn/) and the ACTA2-AS1 binding sites to the miR-4428 was predicted by lncBASE database (http://carolina.imis.athenainnovation.gr/diana_tools/web/index.php?r=lncbasev2/index-predicted) to study the potential crossing network among ACTA2-AS1, miR-4428 and *BCL2L11*.

### Statistical analysis

The data in in study were presented as mean ± standard deviation (SD). The data were visualized through the GraphPad Prism 7.0 software. All statistical analysis was conducted via SPSS 20.0. p < 0.05 presented statistically significant.

## Results

### LncRNA ACTA2-AS1 was down-regulated in COAD tissue samples and cells

**Fig. 1:**
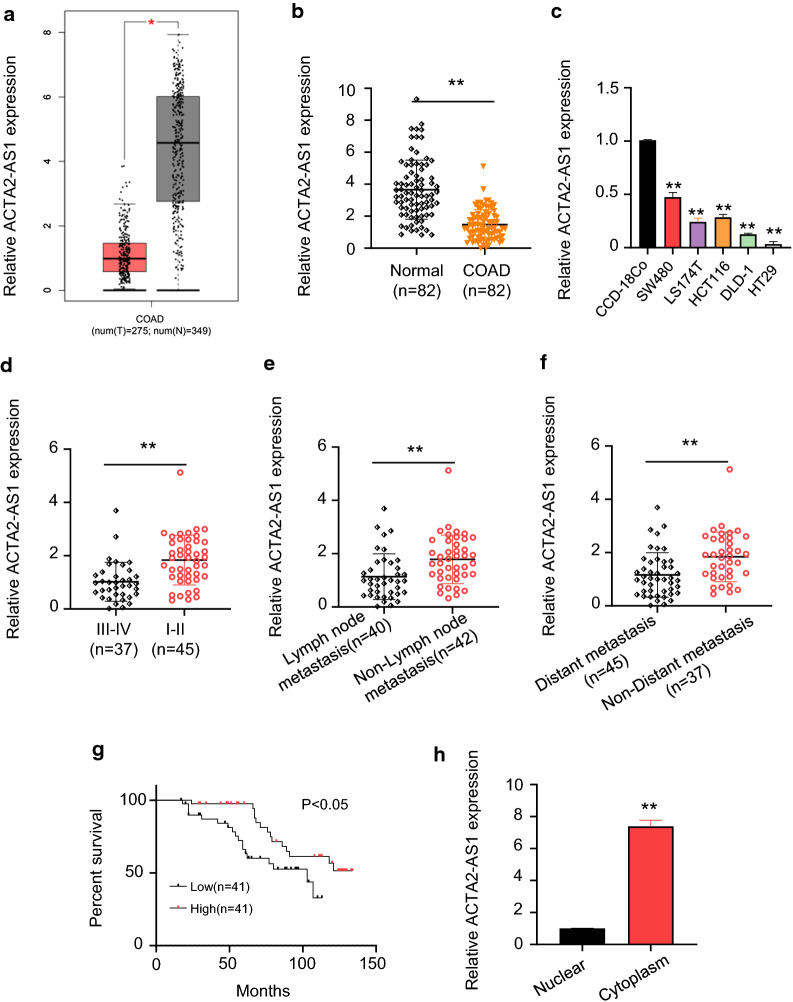
LncRNA ACTA2-AS1 was down-expression in COAD tissue samples and cells. **a** ACTA2-AS1 was down-expressed in COAD samples from TCGA database. **b** Relative ACTA2-AS1 expression in 82 pairs of COAD tissue samples and their adjacent normal tissues using qRT-PCR assay. **c** Relative ACTA2-AS1 expression in different COAD cell lines (SW480, HT29, LS174T, HCT116 and DLD-1) and normal human colon mucosal epithelial cell line CCD-18Co. **d** Relative ACTA2-AS1 expression in different clinical stages of COAD. **e** Downregulation of ACTA2-AS1 in COAD was associated with lymph node metastasis. **f** Downregulation of ACTA2-AS1 in COAD was associated with distant metastasis. **g** The KM-Plotter was used to evaluate survival in COAD patients. Patients with low ACTA2-AS1 expression had worse overall survival than those with high ACTA2-AS1 expression. **h** Localization of ACTA2-AS1 in COAD cells using nuclear cytoplasm separation experiment. *p < 0.05; **p < 0.01

To investigate the role of lncRNA ACTA2-AS1 in COAD tissue samples, we firstly downloaded data from TCGA database and exhibited that the expression of ACTA2-AS1 was obviously upregulated in normal samples compared with COAD samples (Fig. 1a). Then, 82 pairs of human COAD tissue samples and their corresponding adjacent non-tumor samples were detected by qRT-PCR. As shown as Fig. 1b, the expression of ACTA2-AS1 was remarkably low in COAD tissues. In addition, this study detected the expression level of ACTA2-AS1 in six COAD cell lines (SW480, HT29, LS174T, HCT116 and DLD-1) and normal human colon mucosal epithelial cell line CCD-18Co, finding that ACTA2-AS1 expression was extremely lower in COAD cells than in CCD-18Co cell (Fig. 1c). ACTA2-AS1 expression was detected in different clinical stages of COAD, suggesting that the expression of ACTA2-AS1 was negatively correlated with advanced TNM stage. We also observed that ACTA2-AS1 expression was further significantly down-regulated in COAD patients that had lymph node or distal metastasis compared with those without metastasis (Fig. 1e and f). Furthermore, the median expression value of ACTA2-AS1 in COAD tissues was the cut-off value and then divided the tissues into high expression group (n = 41) and low expression group (n = 41), Kaplan–Meier survival analysis suggested that high-ACTA2-AS1 expression was associated with longer survival time in patients with COAD (Fig. 1g). Next, we further demonstrated that ACTA2-AS1 was mainly localized in cytoplasm (Fig. 1h). These results revealed that prominent low-expression of ACTA2-AS1 was detected in COAD samples and low expression of ACTA2-AS1 was related to poor prognosis in patients with COAD (Table 1).

**Table 1:** Relationship between ACTA2-AS1 expression and their clinicopathologic parameters in COAD tissue samples

Variable	ACTA2-AS1 expression		P value
	Low (n = 41)	High (n = 41)	
Age			
< 55	21	18	0.507
≥ 55	20	23	
Gender			
Male	24	26	0.651
Female	17	15	
Distant metastasis			
Negative	10	24	0.002*
Positive	31	17	
Family history			
Yes	19	25	0.184
No	22	16	
TNM stage			
I/II	15	31	< 0.001^*^
III/IV	26	10	
Lymph node metastasis			
Negative	17	32	0.001^*^
Positive	24	9	

### Effect of ACTA2-AS1 on the proliferation and apoptosis of COAD cells

**Fig. 2:**
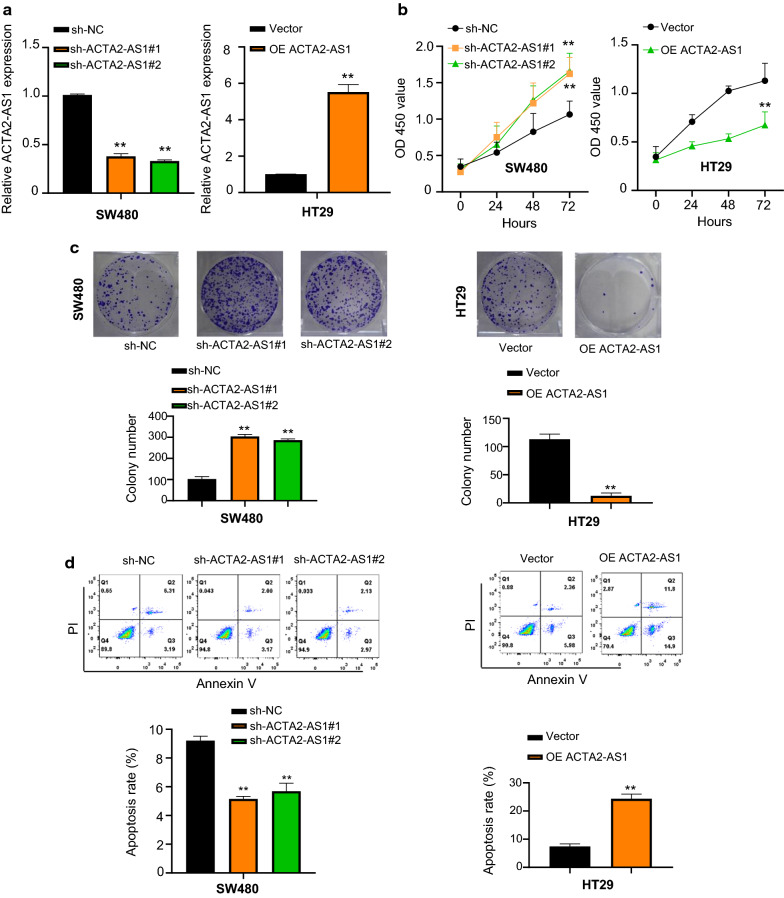
Effect of ACTA2-AS1 on the proliferation and apoptosis of COAD cells. **a** qRT-PCR analysis of knockdown and overexpression efficiency of ACTA2-AS1 in SW480 and HT29 cells transfected with sh-NC, sh-ACTA2-AS1, empty vector and OE ACTA2-AS1 respectively. **b** CCK-8 assay results of cell viability in SW480 and HT29 cells transfected with sh-NC, sh-ACTA2-AS1, empty vector and OE ACTA2-AS1 respectively. **c** Colony formation in SW480 and HT29 cells transfected with sh-NC, sh-ACTA2-AS1, empty vector and OE ACTA2-AS1. **d** Flow cytometric analysis showing the effects of sh-ACTA2-AS1 and OE ACTA2-AS1 on the cell apoptosis in SW480 and HT29 cells. p < 0.05; **p < 0.01

In order to examine the effect of ACTA2-AS1 in COAD, we silenced ACTA2-AS1 expression in SW480 using shRNAs and over-expressed ACTA2-AS1 in HT29 cell lines, respectively. The knockdown and over-expression efficiency results demonstrated that ACTA2-AS1 expression was significantly decreased or increased by sh-ACTA2-AS1 or OE ACTA2-AS1 transfection, respectively (Fig. 2a), suggesting that the effect of ACTA2-AS1 was inhibited or promoted. The results of CCK-8 assay disclosed that knockdown of ACTA2-AS1 obviously increased the proliferative ability of SW480 cells in contrast to the sh-NC groups, while cell proliferation was obviously inhibited in the OE ACTA2-AS1 group compared with vector group (Fig. 2b). Similarly, the clone formation assay revealed that inhibition of ACTA2-AS1 increased the number of colony formation in SW480 cells lines compared with the sh-NC group, while the ability of colony formation was inhibited in the OE ACTA2-AS1 group (Fig. 2c). Next, the apoptosis role of ACTA2-AS1 in COAD cell was detected via flow cytometric analysis assay, indicating that the cell apoptosis rate was obviously decreased or increased after ACTA2-AS1 down-regulation or up-regulation (Fig. 2d). Taken together, lncRNA ACTA2-AS1 could inhibit COAD cell proliferation, and meanwhile promote apoptosis.

### LncRNA ACTA2-AS1 sponged miR-4428 in COAD cells

**Fig. 3:**
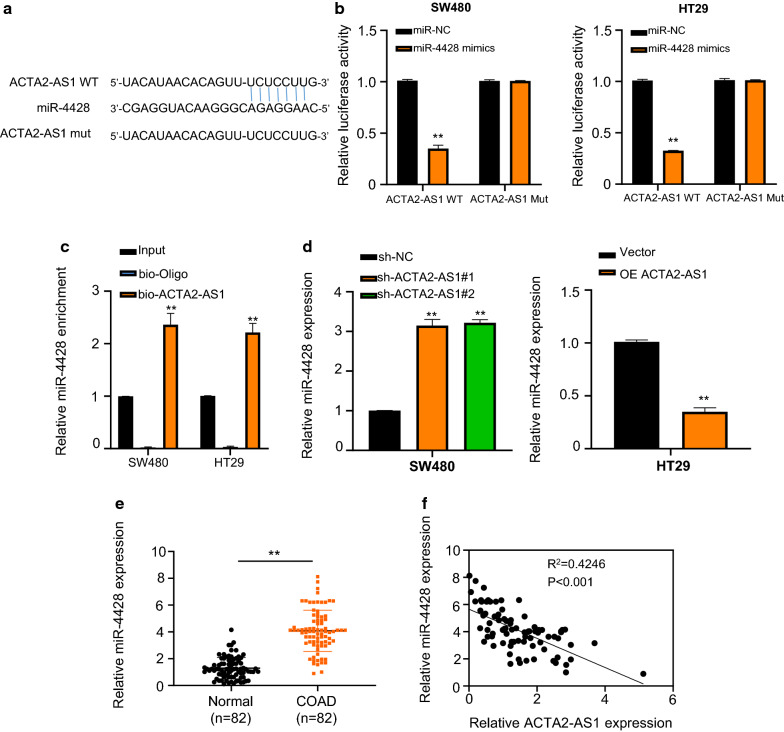
LncRNA ACTA2-AS1 sponged miR-4428 in COAD cells. **a** LncRNA ACTA2-AS1 was predicated to function as molecular sponge for miR-4428 using an online database lncBASE. **b** The putative binding sites between ACTA2-AS1 and miR-4428. Relative miR-4428 expression in cells co-transfected with wt or mut ACTA2-AS1 and miR-4428 in SW480 and HT29 cells by luciferase reporter assay. **c** Enrichment of lncRNA ACTA2-AS1 using RNA pull-down experiments in SW480 and HT29 cells. **d** Relative miR-4428 expression in SW480 and HT29 cells transfected with sh-NC, sh-ACTA2-AS1, empty vector and OE ACTA2-AS1. **e** Relative miR-4428 expression in 82 pairs of COAD tissues and their adjacent normal tissues using qRT-PCR assay. **f** Spearman’s rank-order correlation between miR-4428 and ACTA2-AS1. *p < 0.05; **p < 0.01

To verify the potential molecular target of ACTA2-AS1 involved in COAD cells, an online bioinformatics tool lncBASE was detected. As shown as Fig. 3a, there was a potential complementary sequence between ACTA2-AS1 and miR-4428. Luciferase reporter assay was assessed to detect and verify the interaction of ACTA2-AS1 and miR-4428, finding that the relative luciferase activities were reduced in ACTA2-AS1-wt transfected cells while that in ACTA2-AS1-mut groups displayed no change (Fig. 3b). Moreover, RNA pull-down assay also exhibited that miR-4428 were enriched in bio-ACTA2-AS1 group (Fig. 3c). Transfection of sh-ACTA2-AS1 markedly increased the expression of miR-4428, while transfection of OE ACTA2-AS1 decreased the expression of miR-4428 (Fig. 3d). In addition, qRT-PCR assay also disclosed that miR-4428 was increased in 82 pairs of human COAD tissue comparing with adjacent non-tumor tissues (Fig. 3e). Meanwhile, a negative relationship was observed between the expression of ACTA2-AS1 and miR-4428 in COAD tissues (Fig. 3f). The above results confirmed that ACTA2-AS1 could serve as a sponge of miR-4428 in COAD cells.

### miR-4428 directly interacted with *BCL2L11*

**Fig. 4:**
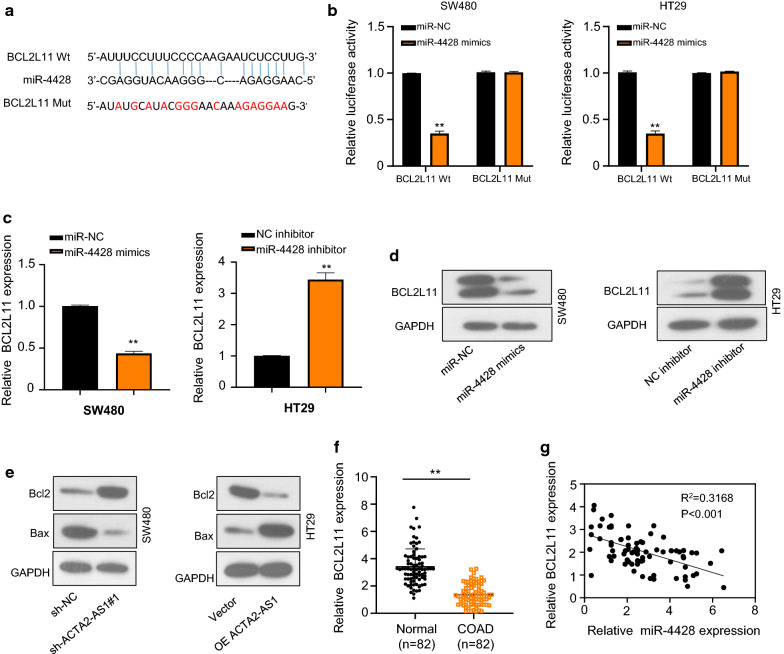
miR-4428 directly interacted with *BCL2L11*. **a** The putative binding sites between *BCL2L11* and miR-4428. **b** Relative *BCL2L11* expression in cells co-transfected with wt and mut *BCL2L11* and miR-4428 mimics in SW480 and HT29 cells using luciferase reporter assay. **c** Relative *BCL2L11* mRNA expression in SW480 and HT29 cells transfected with miR-4428 mimics and miR-4428 inhibitor. **d** Relative *BCL2L11* protein expression in SW480 and HT29 cells transfected with miR-4428 mimics and miR-4428 inhibitor. **e** Apoptosis-related protein Bax and Bcl-2 expression in SW480 and HT29 cells transfected with sh-ACTA2-AS1 and OE ACTA2-AS1. GAPDH was used as a loading control. **f** Relative *BCL2L11* expression in 82 pairs of COAD tissues and their adjacent normal tissues. **g** Spearman’s rank order correlation between miR-4428 and BCL2L1. *p < 0.05; **p < 0.01

To predict the downstream direct target mRNA of miR-4428, an online bioinformatics tool Starbase was carried out. The results showed that an underlying miR-4428 binding sites was detected in the 3ʹ-untranslated region (3ʹUTR) of *BCL2L11* (Fig. 4a). Then, luciferase reporter assay verified that the relative luciferase activities were reduced in *BCL2L11*-wt transfected cells while no significant change of relative luciferase activity was measured in the *BCL2L11*-mut group (Fig. 4b). Data from qRT-PCR and Western blot demonstrated that both mRNA and protein expression level of *BCL2L11* decreased or increased in COAD cell lines transfected with miR-4428 mimics or miR-4428 inhibitor, respectively (Fig. 4c and d). The proteins of apoptosis markers Bcl-2 and Bax were assessed by Western blotting, suggesting that Bcl-2 were up-regulated in the sh-ACTA2-AS1 group in comparison with the control group. However, the level of Bax was suppressed. Next, OE ACTA2-AS1 was transfected into HT29 cells, the expression trend of the above proteins was reversed (Fig. 4e). Conversely, the down-regulation of ACTA2-AS1 resulted in the decrease of Bax when compared with the control transfected cells, while increased in OE ACTA2-AS1 group in comparison with the vector group (Fig. 4e). Next, we identified that *BCL2L11* was memorably down-regulated in 82 pairs of human COAD tissue compared to that in matched adjacent normal tissue (Fig. 4f). And the expression of *BCL2L11* was negatively related miR-4428 expression in human COAD tissues via Spearman’s rank-order analysis (Fig. 4g). These results illustrated that *BCL2L11* directly interacts with miR-4428.

### LncRNA ACTA2-AS1 suppressed COAD progression by sponging miR-4428 upregulation *BCL2L11*

**Fig. 5:**
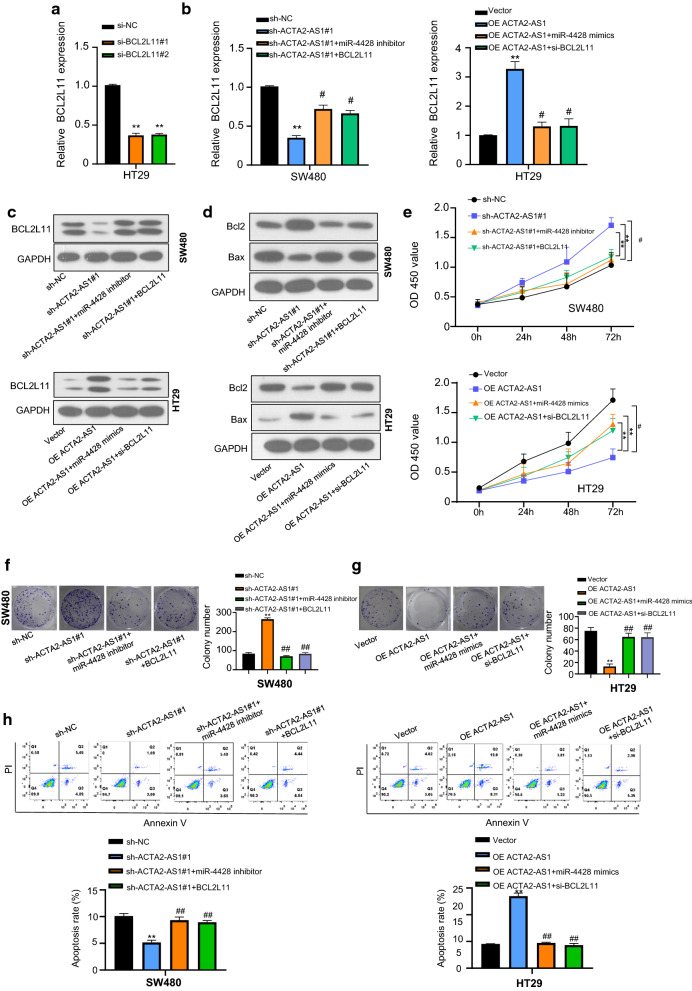
LncRNA ACTA2-AS1 suppressed COAD progression by sponging miR-4428 upregulation *BCL2L11*. **a** Relative *BCL2L11* mRNA expression in HT29 cells transfected with sh-NC and sh- ACTA2-AS1. **b** Relative *BCL2L11* mRNA expression in SW480 transfected with sh-NC, sh-ACTA2-AS1, sh-ACTA2-AS1 + miR-4428 inhibitor and sh-ACTA2-AS1 + *BCL2L11*. And relative *BCL2L11* mRNA expression in HT29 cells transfected with OE ACTA2-AS1, OE ACTA2-AS1 + miR-4428 mimics, and OE ACTA2-AS1 + si-*BCL2L11*, respectively. **c** Relative *BCL2L11* mRNA protein in SW480 transfected with sh-NC, sh-ACTA2-AS1, sh-ACTA2-AS1 + miR-4428 inhibitor and sh-ACTA2-AS1 + *BCL2L11*. And relative *BCL2L11* protein expression in HT29 cells transfected with OE ACTA2-AS1, OE ACTA2-AS1 + miR-4428 mimics, and OE ACTA2-AS1 + si-*BCL2L11*, respectively. **d** Apoptosis-related protein Bax and Bcl-2 expression in SW480 and HT29 cells transfected with sh- ACTA2-AS1, sh-ACTA2-AS1 + miR-4428 inhibitor and sh-ACTA2-AS1 + *BCL2L11*, and OE ACTA2-AS1, OE ACTA2-AS1 + miR-4428 mimics, and OE ACTA2-AS1 + si-*BCL2L11*, respectively. **e** CCK-8 assay of cell viability in SW480 and HT29 cells transfected with sh- ACTA2-AS1, sh-ACTA2-AS1 + miR-4428 inhibitor and sh-ACTA2-AS1 + *BCL2L11*, and OE ACTA2-AS1, OE ACTA2-AS1 + miR-4428 mimics, and OE ACTA2-AS1 + si-*BCL2L11*. **f** Colony formation in SW480 and HT29 cells transfected with sh- ACTA2-AS1, sh-ACTA2-AS1 + miR-4428 inhibitor and sh-ACTA2-AS1 + *BCL2L11*, and OE ACTA2-AS1, OE ACTA2-AS1 + miR-4428 mimics, and OE ACTA2-AS1 + si-*BCL2L11*. **g** Flow cytometric analysis showing the effects of the cell apoptosis in SW480 and HT29 cells transfected with sh-ACTA2-AS1, sh-ACTA2-AS1 + miR-4428 inhibitor and sh-ACTA2-AS1 + *BCL2L11*, and OE ACTA2-AS1, OE ACTA2-AS1 + miR-4428 mimics, and OE ACTA2-AS1 + si-*BCL2L11*, respectively. **p < 0.01

MRNA expression of *BCL2L11* was reduced in HT29 cells by transfection with si-*BCL2L11* (Fig. 5a), and its mRNA and protein expression could be rescued in SW480 cells by sh-ACTA2-AS1 co-transfection with the miR-4428 inhibitor and *BCL2L11*, while the expression of *BCL2L11* was increased by transfection with OE ACTA2-AS1, and its expression could be suppressed in the cells by co-transfection with the miR-4428 mimics and si-*BCL2L11* in HT29 (Fig. 5b–d). Furthermore, both cell proliferation and colony formation were increased in the cells transfected of sh-ACTA2-AS1 in comparison with sh-NC group, while restored in sh-ACTA2-AS1 + miR-4428 inhibitor and sh-ACTA2-AS1 + *BCL2L11* group. Correspondingly, cells transfected with OE ACTA2-AS1, both cell proliferation and colony formation were inhibited compared with sh-NC group and increased by co-transfection with OE ACTA2-AS1 and miR-4428 mimics or OE ACTA2-AS1 and over-expression of *BCL2L11* (Fig. 5e–g). In addition, flow cytometry assays manifested that silence of miR-4428 or over-expression of *BCL2L11* could reverse the promoting apoptosis effect of ACTA2-AS1 (Fig. 5h). These results indicated that ACTA2-AS1 suppressed COAD progression by sponging miR-4428 upregulation *BCL2L11*.

### The suppressive roles of LncRNA ACTA2-AS1 in COAD in vivo experiments

**Fig. 6:**
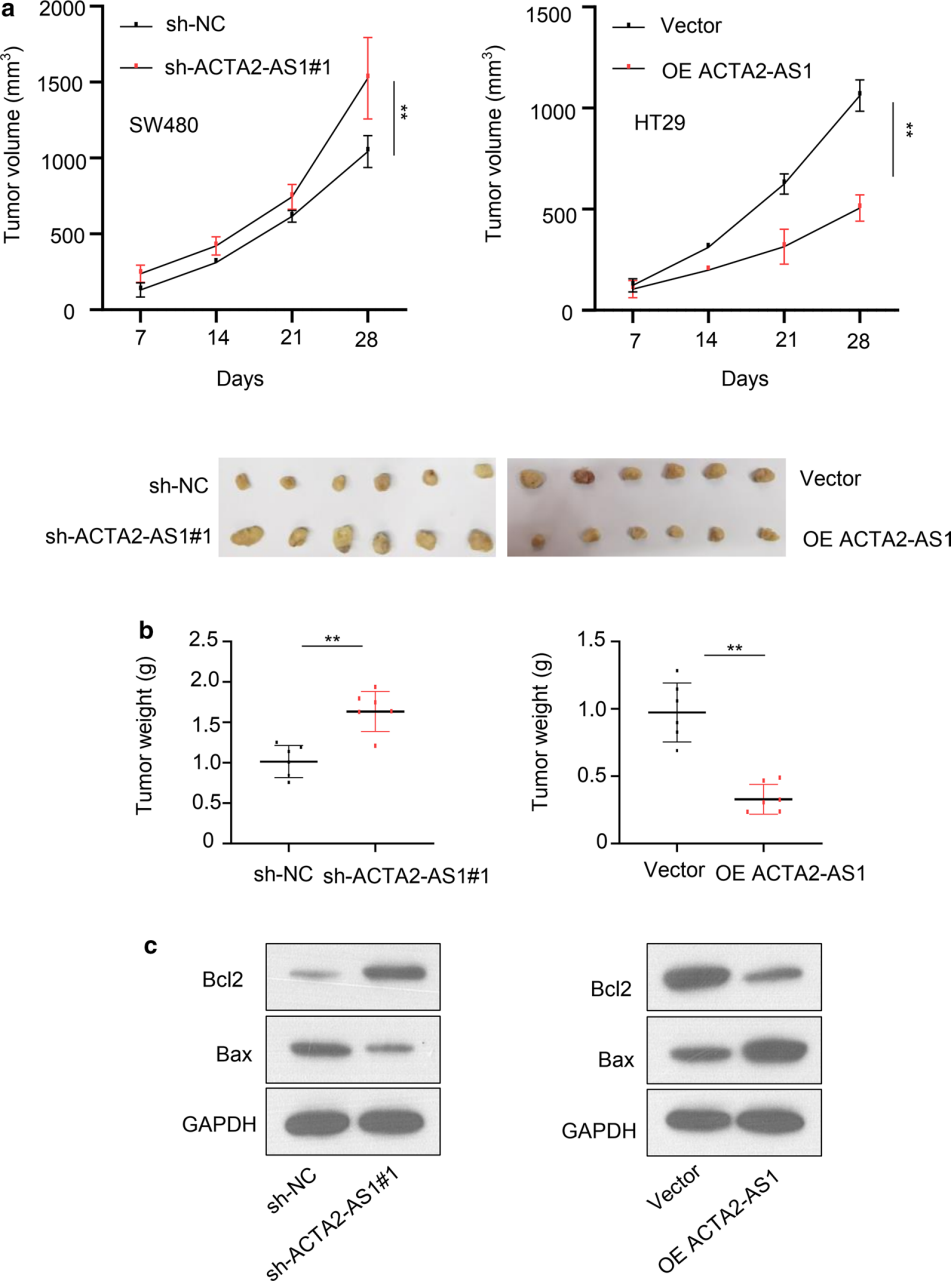
The suppressive roles of LncRNA ACTA2-AS1 in COAD in vivo. **a** Tumor volume and tumor diagram in nude mice implanted subcutaneously with SW480 and HT29 cells transfected with sh-ACTA2-AS1 and lncRNA ACTA2-AS1 OE respectively. **b** Tumor weight in nude mice implanted subcutaneously with SW480 and HT29 cells transfected with sh-ACTA2-AS1 and lncRNA ACTA2-AS1 OE respectively. **c** Apoptosis-related protein Bax and Bcl-2 expression in subcutaneous tumor transfected with sh-ACTA2-AS1 and lncRNA ACTA2-AS1 OE. GAPDH was used as a loading control. *p < 0.05

**Fig. 7:**
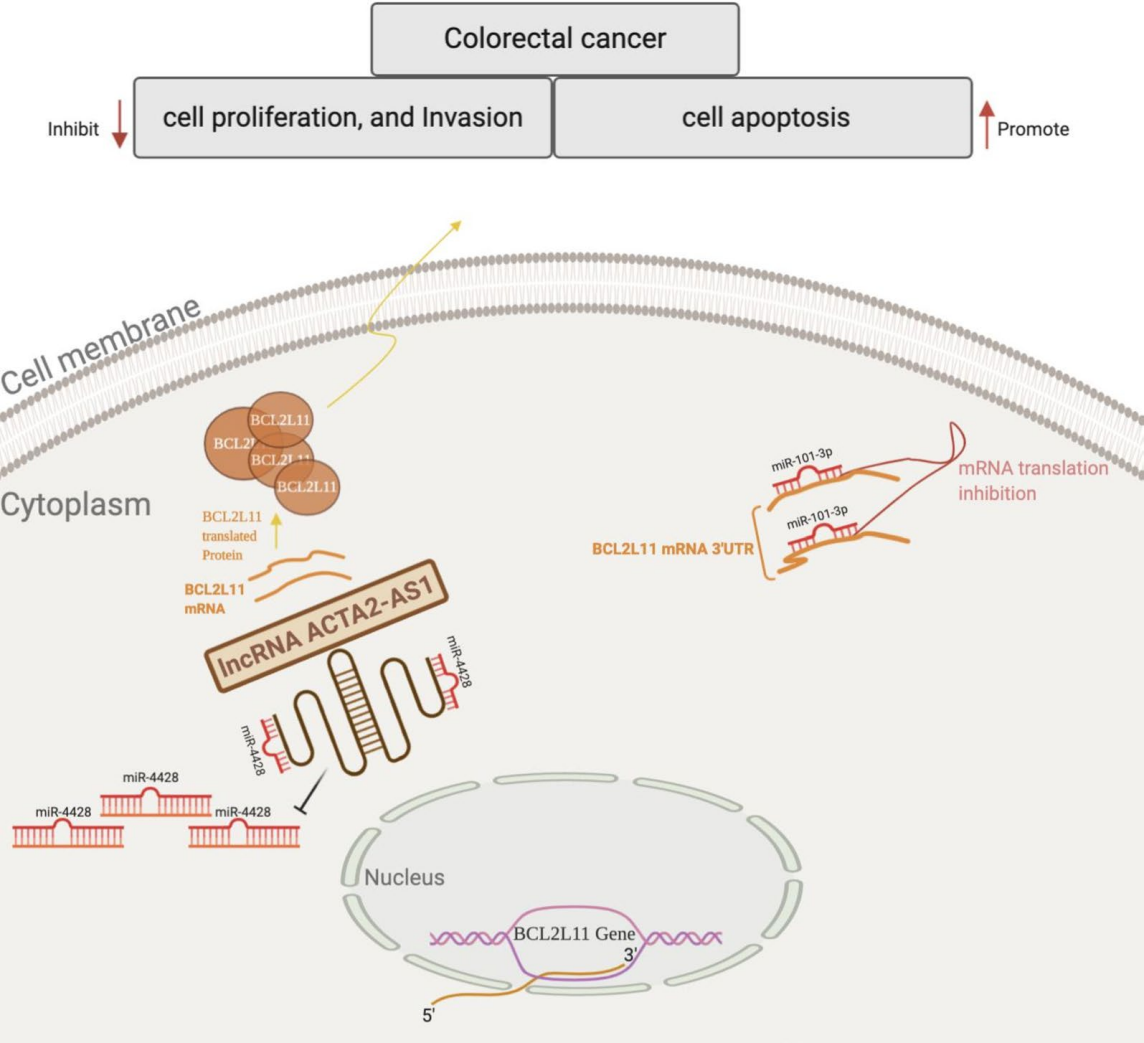
A schematic representation of the ACTA2-AS1 mechanism of regulation of CRC progression

Then, in order to evaluate the effects of ACTA2-AS1 in vivo experiments, the BALB/c nude mice were employed to construct tumor subcutaneous xenografts, the results suggested that the volume and weight of the xenograft tumors were larger or smaller in the sh-ACTA2-AS1 group or in OE ACTA2-AS1 group comparing the sh-NC and vector group respectively (Fig. 6a and b). The proteins of apoptosis markers Bcl-2 and Bax were also assessed in subcutaneous xenograft tumor, the expression of Bcl-2 was showed to significantly increase in sh-ACTA2-AS1 group in comparison with the sh-NC group, while decreased in OE ACTA2-AS1 group in comparison with the vector group. Conversely, the down-regulation of ACTA2-AS1 resulted in the decrease of Bax when compared with the control transfected cells, while restored in OE ACTA2-AS1 group in comparison with the vector group (Fig. 6c). A schematic representation of the ACTA2-AS1 mechanism of regulation of CRC progression is presented in Fig. 7.

## Discussion

It is widely acknowledged that lncRNA could competitively sponge and regulate the expressions of miRNA to regulate tumorigenesis [16–18]. For instance, lncRNA-ROR promotes tumor growth and metastasis of colon cancer cell by targeting miR-145 [19]. In addition, MNX1-AS1 and ELFN1-AS1 were found to facilitate cell proliferation through regulating miR-218-5p/SEC61A1 axis in colon adenocarcinoma [20, 21]. Recent evidence indicated that high-expression of ACTA2-AS1 was positively associated with poor prognosis in COAD patients, accelerating the pathological activities via regulating miR-4644/TRIM44 [22]. LINC00342 and LINC00491 could accelerate progression of COAD by regulating miR-545-5p/MDM2 axis and sponging miR-145 respectively [23, 24].

Recently, it was reported that ACTA2-AS1 is significantly over-expressed in cervical cancer, upregulating SMAD3 expression by competitively sponging miR-143-3p [15]. However, ACTA2-AS1 may act as a tumor suppressor in lung adenocarcinoma and liver cancer cell via sequestering miR-378a-3p and miR-4428 to upregulate the expression of SOX7. However, the role of ACTA2-AS1 in COAD is not completely researched yet. This study suggested that low-expression of ACTA2-AS1 contributing to poor prognosis of COAD through KM-plot analysis. Inhibition or overexpression of ACTA2-AS1 promoted or inhibited cell proliferation, colony formation and induced apoptosis, demonstrating that ACTA2-AS1 might suppress the progress of COAD. Previously, Ying et al. showed that miR-4428 could bind with ACTA2-AS1 and posed positive effects on growth, migration and epithelial-mesenchymal transition process in non-small cell lung cancer [14]. Evidences from our study indicated that miR-4428 could sponge ACTA2-AS1 and regulated the expression of *BCL2L11* negatively. Rescue assays suggested that the impaired COAD cells growth and facilitated apoptosis triggered by over-expression of ACTA2-AS1 could be recovered by knockdown of *BCL2L11* or over-expression of miR-4428.

*BCL2L11* (also known as BIM) is a member of BCL-2 family, inducing apop-tosis and inhibiting autophagy by inactivating BCL2 or by activating BAX-BAK1 and by bridging BECN1 or DYNLL1, respectively [25–27]. According to previous reports, *BCL2L11* is involved in biological pro-cesses in a variety of solid tumors such as ovarian cancer, endometrial adenocarcinoma, prostate tumor and gastric cancer [28–30]. Cumulating evidence has demonstrated that the dysregulation of miRNAs plays crucial roles in the pathology tumorigenesis by directly tar-geting the 3′-UTRs of mRNA of target genes. For example, it is reported that the expression of *BCL2L11* is a direct target of miR-24 in gastric cancer, regulating cell growth and apoptosis. In human endometrial adenocarcinoma, miR-106a mimics co-transfected with wild-type *BCL2L11* 3′-UTR markedly inhibited the relative luciferase activities of RL95-2 and HEC-1B cells, suggesting that *BCL2L11* is the direct target of miR-106a [28]. In this study, we found the 3′-UTRs of *BCL2L11* could sponge miR-4428. Additionally, *BCL2L11* was low-expressed in COAD tissues and negatively correlated with miR-4428, and restoration of *BCL2L11* expression completely rescued the inhibitory effect of up-regulation of ACTA2-AS1 in COAD cells. These results revealed that *BCL2L11* was directly regulated by miR-4428 and might play a crucial role in COAD Additional file 1.

Taken together, ACTA2-AS1 plays a suppressive role in COAD via decoying miR-4428 to augment the expression of *BCL2L11*, inhibiting cell proliferation and promoting apoptosis. Our study demonstrated that ACTA2-AS1 may be a novel prognostic marker and therapeutic target biomarker in COAD.

## Supplementary information

**Additional file 1:** Additional Figures.

## Data Availability

The data and materials have been included in this manuscript, and can contect author with email followed to cite. E-mail: leicj_biology@163.com.
