# Supplementary material for: LncRNA ACTA2-AS1 suppress colon adenocarcinoma progression by sponging miR-4428 upregulation BCL2L11
Source: Cancer Cell Int. 2021 Apr 12;21:203. doi: 10.1186/s12935-021-01769-3 (PMC8042989; doi:10.1186/s12935-021-01769-3)
Supplement: Supplementary file 1 — Additional file 1: Additional Figures. [file 12935_2021_1769_MOESM1_ESM.docx]

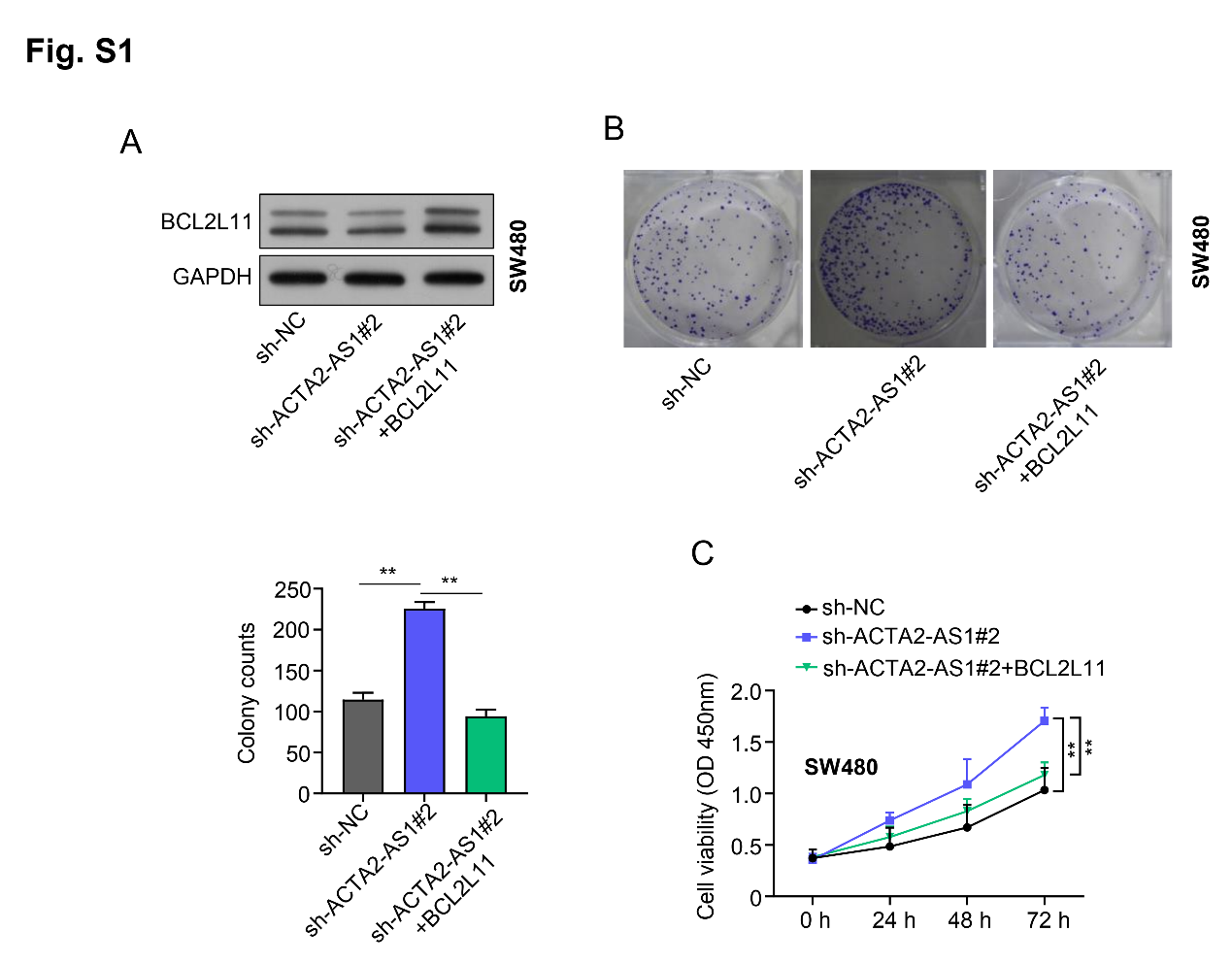


**Fig. S1 The effect of sh-ACTA2-AS1#2 on in vitro experiments.** (A) Relative BCL2L11 protein in SW480 transfected with sh-NC, sh-ACTA2-AS1#2, sh-ACTA2-AS1#2+BCL2L11. (B) Colony formation in SW480 cells transfected with sh-NC, sh-ACTA2-AS1#2 and sh-ACTA2-AS1#2+BCL2L11. (C) CCK-8 assay of cell viability in SW480 cells transfected with sh-NC, sh-ACTA2-AS1#2 and sh-ACTA2-AS1#2+BCL2L11.


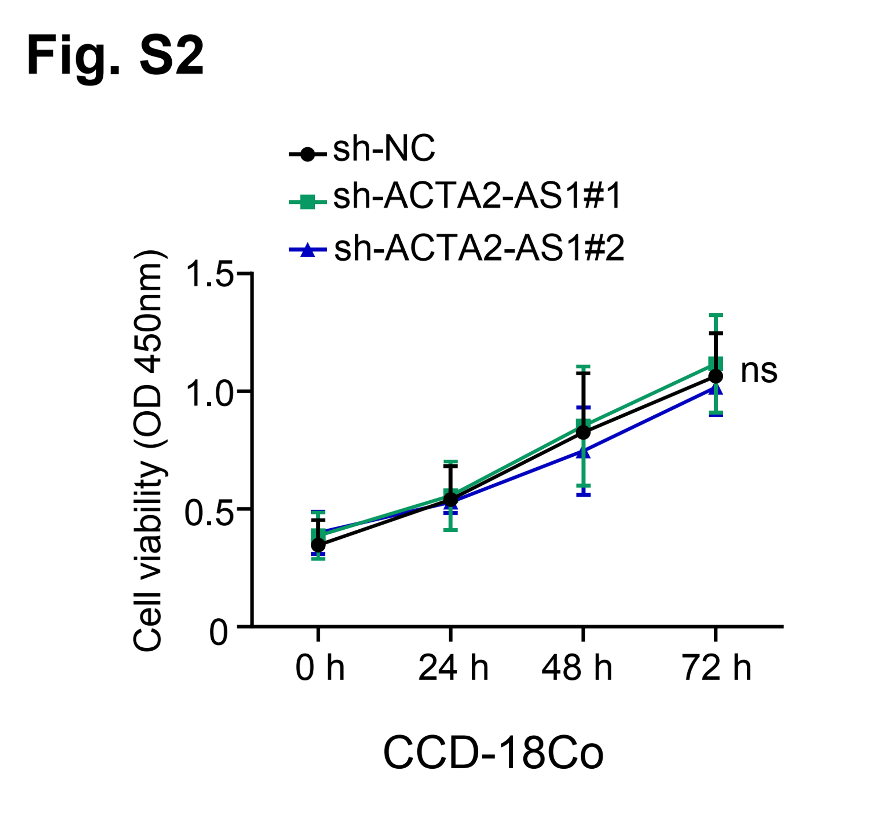


**Fig. S2** CCK-8 assay of cell viability in CCD-18Co cells transfected with sh-NC, sh-ACTA2-AS1#1 and sh-ACTA2-AS1#2.


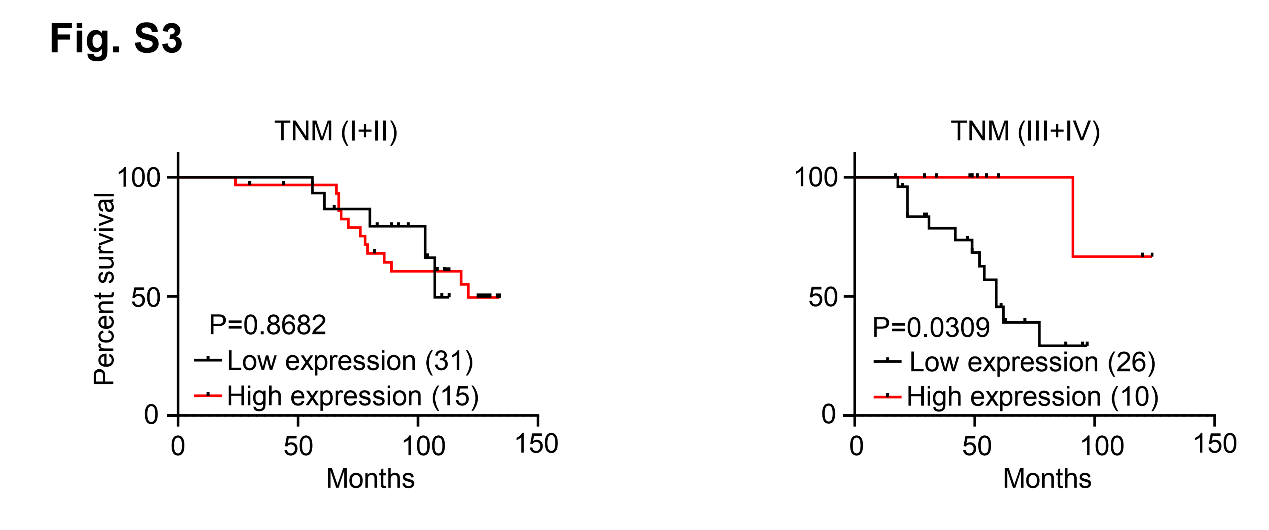


Fig. S3 KM-Plotter was used to evaluate survival in COAD patients with different clinical stages. Low ACTA2-AS1 expression had no obvious change in overall survival of patients with early stage (TNM(I+II)), but low ACTA2-AS1 expression had worse overall survival than those with high ACTA2-AS1 expression with advanced stage (TNM(III+IV)).
